# The Role of Dietary Fiber and Gut Microbiome Modulation in Progression of Chronic Kidney Disease

**DOI:** 10.3390/toxins14030183

**Published:** 2022-03-02

**Authors:** Natarajan Ranganathan, Emmanuel Anteyi

**Affiliations:** Research and Development Lab, Kibow Biotech Inc., 4781 West Chester Pike, Newtown Square, PA 19073, USA

**Keywords:** chronic kidney disease, dietary fiber, gut microbiome

## Abstract

Nutrition is one of the fundamental approaches to promoting and preventing all kinds of diseases, especially kidney diseases. Dietary fiber forms a significant aspect of renal nutrition in treating chronic kidney disease (CKD). Dietary fiber intake influences the composition and metabolism of the gut microbiome with proven roles in reducing uremic toxin production, preserving kidney function, and retarding the progression of CKD through mechanisms of regulating metabolic, immunological, and inflammatory processes. Understanding dietary fiber’s pathogenesis and mechanistic action in modulating host and microbiome interactions provides a potential adjunct therapeutic target for preventing, controlling, and treating CKD patients. In this regard, a recommendation of adequate and appropriate dietary fiber intake to restore beneficial gut microbiota composition would reduce the risks and complications associated with CKD. This mini review summarizes current evidence of the role of dietary fiber intake in modulating the gut microbiome to improve kidney health.

## 1. Introduction

The increasing role and recognition that lifestyle and dietary habits play in the prevention and promotion of disease and health maintenance continue to stimulate research interest to elucidate the course and pathogenesis of clinical conditions such as cardiovascular diseases, obesity, diabetes, cancer, etc., and kidney diseases [1]. While the health benefits of probiotic intervention in chronic kidney disease (CKD) are well known, recent reviews and data on the role of dietary fiber in promoting kidney health are still evolving [2]. This important role of dietary fiber supplementation or naturally occurring foods in promoting health and disease has increased tremendous research interest in gut microbiome modulation for clinical application and potential therapeutic targets by exploring the gut–kidney axis in CKD [2,3]. The importance of nutrition to prevent and slow CKD progression has long been recognized, with traditional and primary approaches of dietary treatment comprising predominantly protein restriction, adequate calorie intake, and correction of electrolytes abnormalities [2,4]. Apart from specific dietary intervention, the role of gut microbiota in reducing uremic toxin production, preserving renal function, and slowing CKD progression has been reported by various studies [4]. High dietary fiber intake modulates the gut microbiome of CKD patients through a complex regulatory effect on host metabolic and immunological processes associated with improved overall health and renal outcomes [4]. This mini-review paper summarizes the critical role dietary fiber intake plays in improving kidney health through gut microbiome modulation and how its clinical application could be a potential cost-effective, add-on treatment to current standard-of-care in patients with CKD.

## 2. What Is Dietary Fiber

**Figure 1:**
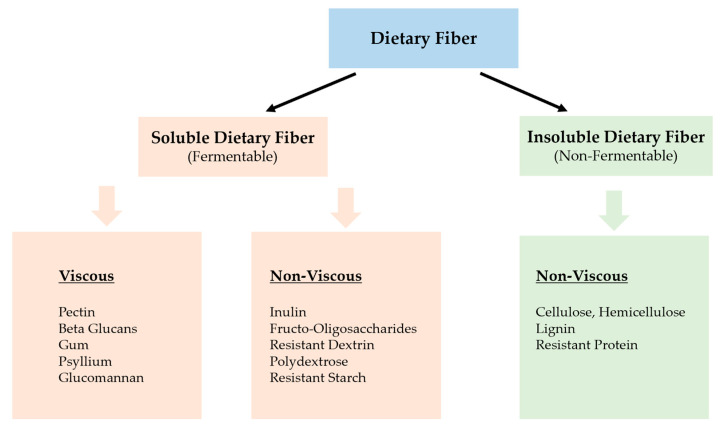
Classification of dietary fibers based on their physicochemical characteristics. (Source: Arranz, S, Remom, AM, Raventro RM et al. (2012). Effects of Dietary Fiber intake on Cardiovascular risk factors. Recent Adv in CVS Risk Factors. Intech open Science/open minds, 978, pp. 59–488) [11].

The term fiber has a broad and flexible meaning depending on the country, food, and pharmaceutical industry definition and classification. Irrespective of its definition, dietary fiber commonly refers to carbohydrates fermentable by gut microbiota providing health benefits to the host. At the same time, the unfermentable remnants serve a bulking or laxation function [4]. Fibers are traditionally classified according to their physicochemical characteristics of solubility; hence, the European Food Safety Authority (EFSA) list of dietary fiber includes undigestible and unabsorbable carbohydrate polymers proven to have scientific evidence of health benefits [5]. Most countries adopted the comprehensive definition of the US Codex Alimentarius commission of 2009, which defined dietary fiber as edible carbohydrate polymers with three or more monomeric units resistant to endogenous digestive enzymes and unabsorbable in small intestines. These edible fibers are further sub-categorized as (i) edible carbohydrate polymers occurring naturally in foods such as fruits, vegetables, legumes, and cereals, (ii) edible carbohydrate polymers obtained from food raw materials by physical, enzymatic, and chemical means with proven physiological benefits, and (iii) synthetic carbohydrate polymers with proven physiological benefit [6,7]. As a result of these diverse sources of dietary fibers, classification based on solubility alone may not predict functional properties since most consumed foods are complex mixes, and cooking may affect the availability of the fiber components [6,7]. Current classification depends on several criteria, including primary food source, chemical structure, water-solubility, viscosity, and fermentability [7]. Based on non-carbohydrate components and monomeric units, dietary fibers are either soluble or insoluble. Fruits and vegetables are rich sources of soluble fibers (pectin, inulin), while wheat bran, oats, and barley have more insoluble dietary fibers of cellulose or hemicellulose (See Figure 1). Gut colon bacteria ferment soluble fibers, with metabolites having beneficial metabolic effects compared to poorly or non-fermentable insoluble fibers with predominant bulking or laxative function in the colon [8]. In addition to solubility and fermentability, the viscosity of soluble fibers such as oligosaccharides influences the absorption of consumed nutrient components in the gut [8,9]. In summary, dietary fiber’s health benefits are attributed to their physicochemical characteristic of water-solubility, viscosity, and fermentability with regulatory effects on body metabolism, obesity, hypertension, cancer, and immunological and inflammatory processes [6,10].

## 3. Health Benefits Effects of Dietary Fiber

The beneficial role of dietary fiber in disease prevention has been partly attributed to its modulation of the gut microbiome in the control of satiety and body weight, regulation of lipid and bile acid metabolism, cancer, and cardiovascular disease risk reduction [12,13,14]. Other known dietary fiber effects on gut microbiota are regulation of inflammation, as observed with decreased C-reactive protein (CRP) marker in an experimental CKD mouse model fed with a high-fat diet following fiber ingestion [15]. Dietary fiber regulates glucose and energy homeostasis through the hypothalamic pathway by modulating gut-derived neuropeptides control of gluconeogenesis in the brain and intestines [16]. In addition, fiber consumption delays gastric emptying and increases satiating hormones, thereby creating a feeling of fullness mediated by the secretion of incretin gut hormone from intestinal L cells [17]. These intestinal incretin hormones are responsible for insulin secretion and glucose homeostasis [17,18]. Other studies have reported an association between high dietary fiber intake and a reduced risk of developing colorectal cancers [19]. All these beneficial effects have been attributed to dietary fiber’s ability to alter gut microbiota in the general population by preventing the occurrence and treating various disease conditions. The challenge with dietary fiber is the lack of a nutritional database to characterize different food sources and quantify fiber classes needed as therapeutic interventions targeting the gut microbiome [20,21].

The recently updated 2020 KDIGO Clinical Practice Guidelines for Nutrition in CKD recommended adequate intake of dietary fiber from natural sources (vegetables and fruits) due to reported decrease in body weight control, blood pressure, and net acid production in CKD stages 3–5 and improvement in lipid profile of post-transplant recipients [22]. Likewise, in diabetic kidney disease, adequate dietary fiber intake in the early stages of CKD was associated with better overall health and renal outcomes [23].

The recommended guideline of fiber intake of a healthy diet in the general population is 20–35 g/day, equivalent to 14 g/1000 kcal [20]. The USA guideline recommends a mean fiber intake of 17 g/day, but only 5% of the population could meet this recommendation. Beyond the total quantity of fiber, the recommendation did not provide further guidance on specific types or proportions of different fiber-containing food required or needed for adequate intake. Noticing this gap has informed some researchers to suggest that any dietary fiber recommendation should include microbiota-accessible carbohydrates components that can be metabolized by colonic microbiota to derive the health benefits. Microbiota-accessible carbohydrates are carbohydrates, whether from plants or animal tissue sources, resistant to digestion and absorption by the host enzymes, and may also be from mucus secretions in the host’s intestine [21].

How dietary fiber contributes to general health has been explained through several mechanisms such as improved colonic transit time, alteration of colon microbial composition and metabolite production, short-chain fatty acids production, and small intestinal lipid and glucose absorption [24]. Short-chain fatty acids (SCFA) production reduces inflammation, alters lipid and glucose metabolism that inhibits carcinogenesis, impacts cardiovascular and metabolic disorders, and potentially reduces the risk and progression of CKD [24,25].

## 4. The Composition of Healthy Gut Microbiota

**Figure 2:**
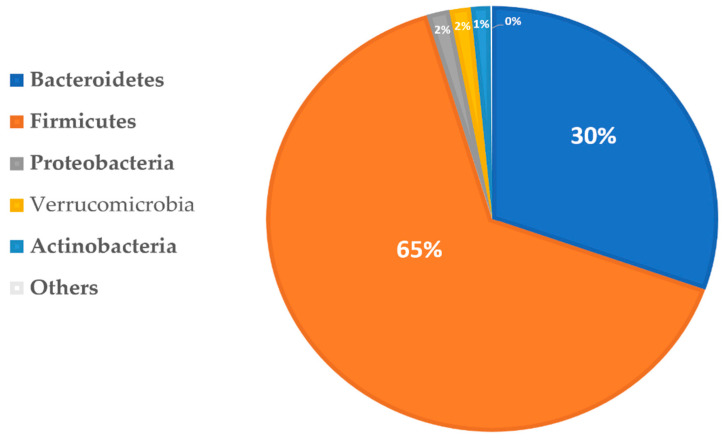
The healthy human gut microbiome phyla classification (source: https://creativecommons.org/licenses/by/4.0/, accessed on 24 February 2022).

The healthy gut microbiota is living microorganisms that form a symbiotic relationship with the host, predominantly bacteria species, including viruses, archaea, fungi, and unicellular eukaryotes [26]. The human gut contains about one trillion of these microorganisms, made of thousands of different species, encoding about three million genes, compared to the human genome of 23,000 genes [27]. Taxonomically, gut microbial bacteria are classified according to phyla, classes, orders, families, genera, and species. There are five phyla in healthy colon microbiota: *Firmicutes* and *Bacteroidetes* (constitute 90%), followed by *Actinobacteria*, *Verrucomicrobia*, and a small proportion of *Proteobacteria* [28]. The Phyla *Firmicutes* have over 200 genera, such as *Clostridium*, *lactobacillus, Enterococcus,* and *Ruminococcus*; the *Bacteroidetes* phyla have predominant genera of *Bacteroides* and *Prevotella,* while The *Bifidobacterium* genus represents actinobacterium phyla [29] (Figure 2). The microbiome composition, diversity, and function differ in everyone depending on various factors such as geographical location, age, sex, race, lifestyle, antibiotics use, and diet. Type of dietary intake is the main factor determining changes in gut microbiome composition, with some of these changes observed as early as a week after switching from a plant to an animal protein-based diet of individuals [30]. The degradation of dietary fibers produces SCFA and vitamins, metabolizes conjugated bile acids, and regulates the immune system [4,30]. The high intake of animal protein-based diet in association with low intake of dietary fiber causes an abundance of *Bacteroidetes* and *Actinobacterium* bacteria that produce trimethylamine (TMA), a precursor of the uremic toxin trimethyl amine-N-oxide (TMAO) that has been implicated in chronic systemic inflammation and cardiovascular disease (CVD) [31,32]. Other health benefits provided to the host by the gut microbiome include the provision of nutrients, protection against pathogenic organisms through competitive exclusion, antimicrobial substances production, development of intestinal mucosa and endothelial integrity, immune modulation, cardiovascular system disease, and cancer risk reduction [29].

## 5. Effect of Dietary Fiber Intake on Gut Microbiome

Dietary fiber administration alters the gut microbial ecosystem by providing substrates for microbial growth and expanding various bacterial species that utilize the different fiber sources. The predominant microbiota composition and changes induced by dietary fiber consumption are specific to the bacterial taxa and species [33]. The gut microbiome degradation capability comprises 130 glycoside hydrolase, 22 polysaccharide lyase, and 16 carbohydrate esterase families, providing the capability to switch between different energy sources of fiber flexibly [7]. The predominant Firmicutes and Actinobacteria species play significant roles in the degradation of complex substrates either as primary or secondary fiber degraders [7]. These observed changes in the fecal microbiome from sustained substrate consumption and subsequent microbial changes are not generally applicable since it depends on individual human host factors [34]. The main pathway of microbial metabolism of dietary fiber is by enzymatic degradation of complex carbohydrates as a source of energy leading to the production of SCFA. The SCFA is composed of acetate, propionate, and butyrate, which regulate the metabolic process, gut mucus production and secretion, immunomodulation, and cell proliferation [35]. Compared to low dietary fiber intake, there is a reduction of microbial diversity and SCFA production, thereby shifting microbial utilization of both dietary and endogenous proteins, including host mucins, resulting in the production of metabolites associated with the development of chronic diseases [6,35]. This beneficial role of high dietary fiber intake was attributed to its promotion of colonic microbial saccharolytic fermentation and, in the process, counteracts the proteolytic fermentation of fats and proteins known to be detrimental to health [6].

Another important health beneficial role of dietary fiber is a contribution to maturation and development of the immune system by the mechanism of SCFA modulation of colonic regulatory T cells through inhibition of histone H3 deacetylase and G protein-coupled receptors (GPR43, GPR41). This immunomodulatory process emanated from the regulatory activity of the innate and adaptive immune systems [6].

Apart from this SCFA-dependent pathway of dietary fiber fermentation, microbial metabolism also occurs through non-SCFA production through bacterial species like *Lactobacillus fermentum* which metabolize cereal bran-based fiber to produce Ferulic acid modulates gut physiology and is found to possess antioxidant and anti-inflammatory properties with potential therapeutic benefits in many chronic diseases [36]. Additionally, dietary fiber can bind different macro-and micronutrients ions like vitamins, copper, calcium, and zinc, transported to the distal gut, released, and absorbed when colonic bacteria metabolize the fiber [37].

## 6. Role of Dietary Fiber in CKD Progression

CKD is a global health problem affecting more than 9% of the world population and a high US prevalence of 15%, associated with high healthcare costs, morbidity, and mortality. The increased risk for CKD death has been attributed to chronic inflammation, oxidative stress, malnutrition, high prevalence of hypertension, diabetes, and CVD. A declining kidney function accumulates uremic retention molecules, notably indoxyl sulfate (IS), p-Cresyl sulfate (PCS), trimethylamine-N-Oxide (TMAO), blood urea nitrogen (BUN), and creatinine, known to be associated with a progressive decline of kidney function, mineral bone disorder, CVD, and increased mortality [38]. The interaction between gut microbiota and CKD is a bidirectional relationship as CKD causes a shift of healthy gut microbiome composition to a state of imbalance between healthy and pathogenic bacteria termed gut dysbiosis. This gut dysbiosis disrupts intestinal epithelial integrity, enhances inflammatory and immunological processes due to endotoxemia, gut-derived uremic toxins, and acidosis which leads to progression and complications [30]. Other contributors to the persistence of dysbiosis include decreased consumption of dietary fibers, frequent antibiotic use, slow colonic transit time, metabolic acidosis, volume overload, intestinal wall edema, and oral iron [30,39]. An experimental finding of dietary fiber effect on dysbiosis in mouse CKD model fed with high amylose resistant starch diet showed decreased microbial diversity, increased ratio of beneficial *Bifidobacteria* genera and *Bacteroidetes* to *Firmicutes* phyla [39]. A similar finding in human CKD stage 3–4 showed increased *Bifidobacteria* and *Lactobacillus* species counts after short-term lactulose supplementation [40]. Carefully selected dietary fiber administration was found appropriate and effective in reducing uremic toxins in CKD patients with fibers containing resistant starch, arabino-xylo-oligosaccharide, gum acacia, and Xylo-oligosaccharide [41]. In another study of hemodialysis (HD) patients, resistant starch supplementation reduced plasma indoxyl sulfate (IS) levels, while an oligofructose enriched inulin diet reduced the serum PCS levels [42]. These observations on reducing plasma levels of uremic toxins following interventions with different dietary fiber types were attributed to differences in the effectiveness of modulating gut microbiome to produce sufficient SCFA to restore gut barrier integrity [43]. Hence, studies have recommended that CKD patients should increase adequate consumption of dietary multifiber and vegetable-based diet to restore intestinal integrity, improve metabolic profile, prevent comorbidities, and retard CKD progression [44]. Due to diet restrictions in CKD patients, supplementary dietary multifiber is a supportive nutritional therapy in all CKD stages to enhance the removal of uremic toxins linked to cardiovascular complications [45]. High fiber intake as a recommended renal diet in CKD remains a challenge due to concerns of high potassium and phosphorus levels. To overcome these fears, fiber from natural foods sources (fruits, vegetables, whole grains) as plant-based diets should be preferred due to better nutrient composition and relatively lower bioavailability of potassium and phosphorus [46].

## 7. Dietary Fiber in Renal Diet

The traditional diet plan for CKD patients comprises restricted protein intake and increased complex carbohydrates with a preference for plant rather than animal sources of these foods [46,47]. These complex fiber-rich carbohydrates with a low protein diet favor a healthy gut microbiota composition, resulting in metabolism and reducing nitrogenous waste retention products [47]. Dietary fiber is a substrate for saccharolytic fermentation that produces SCFA, which has an anti-inflammatory function, regulates immune function, and preserves intestinal barrier integrity. Blood urea nitrogen (BUN) which diffuses from the systemic circulation into the intestinal lumen is hydrolyzed to ammonium hydrochloride by urease-positive species and causes intestinal epithelial damage, increases intestinal permeability, and thus diffusion of bacterial toxins to the bloodstream [48]. Consequently, triggers local and systemic inflammation, leading to further intestinal epithelial damage, and progression of renal impairment [49].

In contrast, animal-based components of the renal diet promote proteolytic fermentation products like phenols, indole, amines, and ammonia which are toxic metabolites found to reduce SCFA levels and thereby counters the beneficial role in the maintenance of intestinal integrity [49,50]. Prebiotic dietary fiber-containing fructooligosaccharide (FOS) and the hydrolyzed product of Inulin have been used to modulate gut microbiome by promoting the growth of beneficial species like *Bifidobacterium* and *Lactobacillus* via the saccharolytic metabolic pathway [50]. A restricted intake of a protein diet in pre-dialysis stages can cause malnutrition. Dietary fiber supplementation with added essential amino acids is recommended to reduce uremic toxins and reduce mortality and morbidity in CKD. In the case of dialysis patients, a plant-based diet with most protein requirement from plant sources which also provides dietary fiber is preferable to avoid malnutrition, rather than the usual recommended high animal protein intake, which increases the production of gut-derived uremic toxins (IS, PCS, TMAO) with associated high cardiovascular mortality [51]. Studies have shown that increased dietary fiber of oligosaccharide-rich Inulin intake for four weeks in hemodialysis patients reduced PCS levels due to SCFA producing microorganisms [52]. Similar findings were seen in non-diabetic peritoneal dialysis, where supplementary dietary fiber intake reduced mortality and cardiovascular complications [53]. Recent reviews of plant-based dietary fiber foods have shown its beneficial role in primary and secondary prevention of CKD, reduced CKD complications of hyperphosphatemia, hypertension, metabolic acidosis, uremic toxemia, hyperlipidemia, diabetes, kidney stones, and all-cause mortality [54].

Renal diets fortified with dietary fiber will improve the current low fiber intake in CKD/ESRD patients from 11–12 g/day to the recommended 25 g/day for women and 38 g/day for men, which is equivalent to 14 g/1000 kcal/day recommended for the general population [55].

## 8. Conclusions

**Figure 3:**
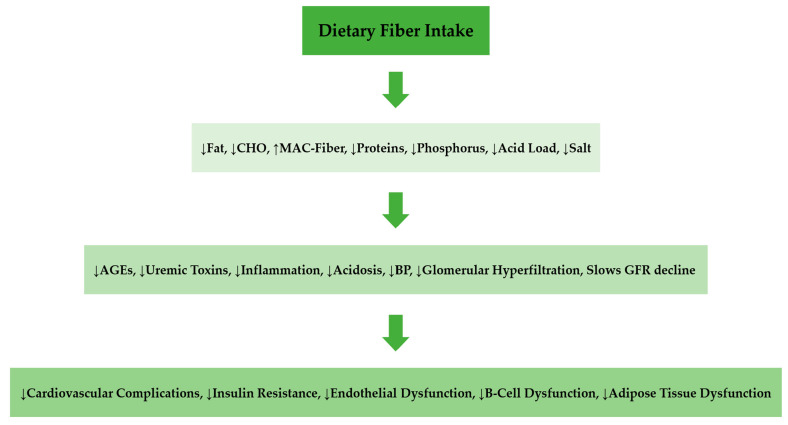
Possible effects of dietary fiber on gut microbiome to slow progression of CKD. Key: AGEs—advanced glycation end products; BP—blood pressure; CHO—carbohydrate; GFR—glomerular filtration rate; MAC-fiber—microbiome-accessible carbohydrate fiber.

Adequate dietary fiber intake promotes good health, prevents and retards diverse chronic diseases, particularly chronic kidney disease. Increased fiber food intake or fiber supplementation reduces the risks and progression of CKD like hypertension, diabetes, obesity, dyslipidemia, and the development of colon cancer [56]. Recognition of dietary fiber either as prebiotic supplements or naturally occurring foods in health and disease has equally increased general public interest as published in the science report of The New York Times article on benefits of fiber-rich foods has created the urgent need for more research in the field of gut microbiome modulation [57]. These health benefits relate to its effect on gut microbiome modulation to regulate the host metabolism, energy homeostasis, and immune system. The primary mechanism of action is to promote gut health through balanced immune function, intestinal mucus production, and membrane integrity, thereby preventing pathogenic microbial growth and disease. The main beneficial effect of dietary fiber intake is gut microbiome modulation through intestinal fermentation and SCFA production. The SCFA, through cellular and signaling mechanisms, plays significant roles in regulating the host metabolic, immunological, and inflammatory processes. The impact of fermentable fiber intake is the correction of CKD-associated dysbiosis, which favors the growth of beneficial gut bacteria, reduces gut-derived uremic toxins, inflammation, and oxidative stress, and improves metabolic profile, resulting in retarding progression CKD and development of CKD-associated comorbidities (See Figure 3).This review has shown the emerging role dietary fiber plays in preventive and promotive health in the context of chronic kidney dysfunction through the gut microbiota modulation pathway. This is a potentially cost-effective approach for conservatively managing patients in advanced CKD stages who are clinically unsuitable or declined renal replacement options. The future challenge is screening and identifying novel fibers from dietary, modified, or synthetic sources that could be formulated as prebiotics or Synbiotic targeted at specific gut microbiota associated with chronic kidney diseases as add-on therapy to standard of care. Hence, Zhao et al. [58] in a recent review, suggested that a comprehensive approach to these specific gut microbiota alterations in CKD is to characterize the diversity, bacterial taxa, gut derived metabolites and gut permeability that could help identify contributors to CKD pathogenesis or progression. Further studies are required to explore complex interactions between specific dietary fiber and gut microbiota to develop personalized nutritional therapy for individuals at reducing the incidence and impact of chronic kidney disease.

## Data Availability

The data presented in this study are available in this article.

## References

[1] Mandaliya D., Patel S., Seshadri S. (2018). Fiber in our diet and its role in health and disease. Functional Food and Human Health.

[2] Anderson J.W., Baird P., Davis R.H., Ferreri S., Knudtson M., Koraym A., Williams C.L. (2009). Health benefits of dietary fiber. Nutr. Rev..

[3] Khoury T., Tzukert K., Abel R., Abu Rmeileh A., Levi R., Ilan Y. (2017). The gut-kidney axis in chronic renal failure: A new potential target for therapy. Hemodial. Int..

[4] Smith C.E., Tucker K.L. (2011). Health benefits of cereal fiber: A review of clinical trials. Nutr. Res. Rev..

[5] Su G., Qin X., Yang C., Sabatino A., Kelly J.T., Avesani C.M., Carrero J.J. (2021). Fiber intake and health in people with chronic kidney disease. Clin. Kidney J..

[6] Makki K., Deehan E.C., Walter J., Bäckhed F. (2018). The impact of dietary fiber on gut microbiota in host health and disease. Cell Host Microbe.

[7] Deehan E.C., Duar R.M., Armet A.M., Perez-Muñoz M.E., Jin M., Walter J. (2017). Modulation of the gastrointestinal microbiome with nondigestible fermentable carbohydrates to improve human health. Microbiol. Spectr..

[8] Cronin P., Joyce S.A., O’Toole P.W., O’Connor E.M. (2021). Dietary Fiber Modulates the Gut Microbiota. Nutrients.

[9] Lovegrove A., Edwards C.H., De Noni I., Patel H., El S.N., Grassby T., Shewry P.R. (2017). Role of polysaccharides in food, digestion, and health. Crit. Rev. Food Sci. Nutr..

[10] Ma Y., Griffith J.A., Chasan-Taber L., Olendzki B.C., Jackson E., Stanek E.J., Ockene I.S. (2006). Association between dietary fiber and serum C-reactive protein. Am. J. Clin. Nutr..

[11] Arranz S., Remon A.M., Raventos R.M., Estruch R.L. (2021). Effects of dietary fiber intake on cardiovascular risk factors. Recent Advances in Cardiovascular Risk Factors.

[12] Othman R.A., Moghadasian M.H. (2011). Beyond cholesterol-lowering effects of plant sterols: Clinical and experimental evidence of anti-inflammatory properties. Nutr. Rev..

[13] Tuan J., Chen Y.X. (2016). Dietary and lifestyle factors associated with colorectal cancer risk and interactions with microbiota: Fiber, red or processed meat and alcoholic drinks. Gastrointest. Tumors.

[14] Rajput P., Prajapati B., kumar Jena P., Seshadri S. The role of gut microbiota produced Short Chain Fatty Acids (SCFAs) in adiposity and inflammation in obesity and type 2 Diabetes. Proceedings of the 6th World Congress of Biotechnology.

[15] Mattace Raso G., Simeoli R., Russo R., Iacono A., Santoro A., Paciello O., Meli R. (2013). Effects of sodium butyrate and its synthetic amide derivative on liver inflammation and glucose tolerance in an animal model of steatosis induced by high fat diet. PLoS ONE.

[16] De Vadder F., Kovatcheva-Datchary P., Goncalves D., Vinera J., Zitoun C., Duchampt A., Mithieux G. (2014). Microbiota-generated metabolites promote metabolic benefits via gut-brain neural circuits. Cell.

[17] Anderson J.W., Pasupuleti V., Anderson J. (2008). Dietary fiber and associated phytochemicals in prevention and reversal of diabetes. Nutraceuticals Glycemic Health Type 2 Diabetes.

[18] Frost G., Sleeth M.L., Sahuri-Arisoylu M., Lizarbe B., Cerdan S., Brody L., Bell J.D. (2014). The short-chain fatty acid acetate reduces appetite via a central homeostatic mechanism. Nat. Commun..

[19] O’Keefe S.J., Li J.V., Lahti L., Ou J., Carbonero F., Mohammed K., Zoetendal E.G. (2015). Fat, fibre and cancer risk in African Americans and rural Africans. Nat. Commun..

[20] Gill S.K., Rossi M., Bajka B., Whelan K. (2021). Dietary fiber in gastrointestinal health and disease. Nat. Rev. Gastroenterol. Hepatol..

[21] Patterson M.A., Maiya M., Stewart M.L. (2020). Resistant starch content in foods commonly consumed in the United States: A narrative review. J. Acad. Nutr. Diet..

[22] Kizler T.A., Burrowes J.D., Byham-Gray L.D., Campbell K.L., Carrero J.J., Chan W., Cuppari L. (2020). KDOQI clinical practice guideline for nutrition in CKD: 2020 update. Am. J. Kidney Dis..

[23] De Boer I.H., Caramori M.L., Chan J.C., Heerspink H.J., Hurst C., Khunti K., Rossing P. (2020). KDIGO 2020 clinical practice guideline for diabetes management in chronic kidney disease. Kidney Int..

[24] Tan J., McKenzie C., Potamitis M., Thorburn A.N., Mackay C.R., Macia L. (2014). The role of short-chain fatty acids in health and disease. Adv. Immunol..

[25] Yang H.L., Feng P., Xu Y., Hou Y.Y., Ojo O., Wang X.H. (2021). The Role of Dietary Fiber Supplementation in Regulating Uremic Toxins in Patients with Chronic Kidney Disease: A Meta-Analysis of Randomized Controlled Trials. J. Ren. Nutr..

[26] Kim S.M., Han Song I. (2020). The clinical impact of gut microbiota in chronic kidney disease. Korean J. Intern. Med..

[27] Rooks M.G., Garrett W.S. (2016). Gut microbiota, metabolites and host immunity. Nat. Rev. Immunol..

[28] Eckburg P.B., Bik E.M., Bernstein C.N., Purdom E., Dethlefsen L., Sargent M., Relman D.A. (2005). Diversity of the human intestinal microbial flora. Science.

[29] Rinninella E., Raoul P., Cintoni M., Franceschi F., Miggiano G., Gasbarrini A., Mele M.C. (2019). What is the Healthy Gut Microbiota Composition? A Changing Ecosystem across Age, Environment, Diet, and Diseases. Microorganisms.

[30] Kanbay M., Onal E.M., Afsar B., Dagel T., Yerlikaya A., Covic A., Vaziri N.D. (2018). The crosstalk of gut microbiota and chronic kidney disease: Role of inflammation, proteinuria, hypertension, and diabetes mellitus. Int. Urol. Nephrol..

[31] Dominguez-Bello M.G., Blaser M.J., Ley R.E., Knight R. (2011). Development of the human gastrointestinal microbiota and insights from high-throughput sequencing. Gastroenterology.

[32] Cho I., Blaser M.J. (2012). The human microbiome: At the interface of health and disease. Nat. Rev. Genet..

[33] Walker A.W., Ince J., Duncan S.H., Webster L.M., Holtrop G., Ze X., Flint H.J. (2011). Dominant and diet-responsive groups of bacteria within the human colonic microbiota. ISME J..

[34] Martínez I., Kim J., Duffy P.R., Schlegel V.L., Walter J. (2010). Resistant starch types 2 and 4 have differential effects on the composition of the fecal microbiota in human subjects. PLoS ONE.

[35] Koh A., De Vadder F., Kovatcheva-Datchary P., Bäckhed F. (2016). From dietary fiber to host physiology: Short-chain fatty acids as key bacterial metabolites. Cell.

[36] Tomaro-Duchesneau C., Saha S., Malhotra M., Coussa-Charley M., Kahouli I., Jones M.L., Prakash S. (2012). Probiotic ferulic acid esterase active Lactobacillus fermentum NCIMB 5221 APA microcapsules for oral delivery: Preparation and in vitro characterization. Pharmaceuticals.

[37] Baye K., Guyot J.P., Mouquet-Rivier C. (2017). The unresolved role of dietary fibers on mineral absorption. Crit. Rev. Food Sci. Nutr..

[38] Kim K.M., Oh H.J., Choi H.Y., Lee H., Ryu D.R. (2019). Impact of chronic kidney disease on mortality: A nationwide cohort study. Kidney Res. Clin. Pract..

[39] Mafra D., Borges N., Alvarenga L., Esgalhado M., Cardozo L., Lindholm B., Stenvinkel P. (2019). Dietary components that may influence the disturbed gut microbiota in chronic kidney disease. Nutrients.

[40] Tayebi-Khosroshahi H., Habibzadeh A., Niknafs B., Ghotaslou R., Sefidan F.Y., Ghojazadeh M., Parkhide S. (2016). The effect of lactulose supplementation on fecal microflora of patients with chronic kidney disease; a randomized clinical trial. J. Ren. Inj. Prev..

[41] Poesen R., Evenepoel P., de Loor H., Delcour J.A., Courtin C.M., Kuypers D., Meijers B. (2016). The influence of prebiotic arabinoxylan oligosaccharides on microbiota derived uremic retention solutes in patients with chronic kidney disease: A randomized controlled trial. PLoS ONE.

[42] Meijers B.K., De Preter V., Verbeke K., Vanrenterghem Y., Evenepoel P. (2010). p-Cresyl sulfate serum concentrations in hemodialysis patients are reduced by the prebiotic oligofructose-enriched inulin. Nephrol. Dial. Transplant..

[43] pChiavaroli L., Mirrahimi A., Sievenpiper J.L., Jenkins D.J.A., Darling P.B. (2015). Dietary fiber effects in chronic kidney disease: A systematic review and meta-analysis of controlled feeding trials. Eur. J. Clin. Nutr..

[44] Markowiak P., Śliżewska K. (2017). Effects of probiotics, prebiotics, and synbiotics on human health. Nutrients.

[45] D’Alessandro C., Piccoli G.B., Calella P., Brunori G., Pasticci F., Egidi M.F., Cupisti A. (2016). “Dietaly”: Practical issues for the nutritional management of CKD patients in Italy. BMC Nephrol..

[46] Joshi S., McMacken M., Kalantar-Zadeh K. (2021). Plant-based diets for kidney disease: A guide for clinicians. Am. J. Kidney Dis..

[47] Ramezani A., Raj D.S. (2014). The gut microbiome, kidney disease, and targeted interventions. J. Am. Soc. Nephrol..

[48] Lau W.L., Vaziri N.D. (2017). The leaky gut and altered microbiome in chronic kidney disease. J. Ren. Nutr..

[49] Hobby G.P., Karaduta O., Dusio G.F., Singh M., Zybailov B.L., Arthur J.M. (2019). Chronic kidney disease and the gut microbiome. Am. J. Physiol. Ren. Physiol..

[50] Koppe L., Fouque D., Soulage C.O. (2018). The role of gut microbiota and diet on uremic retention solutes production in the context of chronic kidney disease. Toxins.

[51] Garneata L., Stancu A., Dragomir D., Stefan G., Mircescu G. (2016). Ketoanalogue-supplemented vegetarian very low–protein diet and CKD progression. J. Am. Soc. Nephrol..

[52] Sirich T.L., Plummer N.S., Gardner C.D., Hostetter T.H., Meyer T.W. (2014). Effect of increasing dietary fiber on plasma levels of colon-derived solutes in hemodialysis patients. Clin. J. Am. Soc. Nephrol..

[53] Xu X., Li Z., Chen Y., Liu X., Dong J. (2019). Dietary fibre and mortality risk in patients on peritoneal dialysis. Br. J. Nutr..

[54] Adair K.E., Bowden R.G. (2020). Ameliorating chronic kidney disease using a whole food plant-based diet. Nutrients.

[55] King D.E., Mainous III A.G., Lambourne C.A. (2012). Trends in dietary fiber intake in the United States, 1999–2008. J. Acad. Nutr. Diet..

[56] Camerotto C., Cupisti A., D’Alessandro C., Muzio F., Gallieni M. (2019). Dietary fiber and gut microbiota in renal diets. Nutrients.

[57] Zimmer C. (2018). Fiber Is Good for You. Now Scientists May Know Why. The New York Times.

[58] Zhao J., Ning X., Liu B., Dong R., Bai M., Sun S. (2021). Specific alterations in gut microbiota in patients with chronic kidney disease: An updated systematic review. Ren. Fail..

